# Transplanted human photoreceptors transfer cytoplasmic material but not to the recipient mouse retina

**DOI:** 10.1186/s13287-024-03679-3

**Published:** 2024-03-14

**Authors:** Margaret T. Ho, Kotoe Kawai, Dhana Abdo, Lacrimioara Comanita, Arturo Ortin-Martinez, Yui Ueno, Emily Tsao, Azam Rastgar-Moghadam, Chang Xue, Hong Cui, Valerie A. Wallace, Molly S. Shoichet

**Affiliations:** 1 Institute of Biomedical Engineering, University of Toronto, Toronto, ON Canada; 2 Terrence Donnelly Centre for Cellular and Biomolecular Research, University of Toronto, 160 College Street, Toronto, ON M5S 3E2 Canada; 3 Donald K. Johnson Eye Institute, Krembil Research Institute, University Health Network, 60 Leonard Ave, Toronto, ON M5T 2S8 Canada; 4 Department of Chemical Engineering and Applied Chemistry, University of Toronto, Toronto, ON Canada; 5 Regenerative Medicine Research and Planning Division, Rohto Pharmaceutical Co., Ltd., 6-5-4 Kunimidai, Kizugawa, Kyoto 619-0216 Japan; 6 Department of Laboratory Medicine and Pathobiology, University of Toronto, Toronto, ON Canada; 7 Department of Ophthalmology and Vision Sciences, University of Toronto, Toronto, ON Canada; 8 Department of Chemistry, University of Toronto, Toronto, ON Canada

**Keywords:** Material transfer, Human retinal organoids, Photoreceptor transplantation

## Abstract

**Background:**

The discovery of material transfer between transplanted and host mouse photoreceptors has expanded the possibilities for utilizing transplanted photoreceptors as potential vehicles for delivering therapeutic cargo. However, previous research has not directly explored the capacity for human photoreceptors to engage in material transfer, as human photoreceptor transplantation has primarily been investigated in rodent models of late-stage retinal disease, which lack host photoreceptors.

**Methods:**

In this study, we transplanted human stem-cell derived photoreceptors purified from human retinal organoids at different ontological ages (weeks 10, 14, or 20) into mouse models with intact photoreceptors and assessed transfer of human proteins and organelles to mouse photoreceptors.

**Results:**

Unexpectedly, regardless of donor age or mouse recipient background, human photoreceptors did not transfer material in the mouse retina, though a rare subset of donor cells (< 5%) integrated into the mouse photoreceptor cell layer. To investigate the possibility that a species barrier impeded transfer, we used a flow cytometric assay to examine material transfer in vitro*.* Interestingly, dissociated human photoreceptors transferred fluorescent protein with each other in vitro, yet no transfer was detected in co-cultures of human and mouse photoreceptors, suggesting that material transfer is species specific.

**Conclusions:**

While xenograft models are not a tractable system to study material transfer of human photoreceptors, these findings demonstrate that human retinal organoid-derived photoreceptors are competent donors for material transfer and thus may be useful to treat retinal degenerative disease.

**Supplementary Information:**

The online version contains supplementary material available at 10.1186/s13287-024-03679-3.

## Background

Photoreceptor degeneration is irreversible due to the limited regenerative capacity of the mammalian central nervous system. Cell therapy to the retina has been an ongoing pursuit since the 1980s with the hope that the new healthy donor cells deposited in the subretinal space would engraft and form new synaptic connections with the host retina to restore vision [1, 2]. The amount of GFP-labelled donor cells found inside the mouse outer nuclear layer (ONL), termed cellular integration, was used to evaluate transplant success. In 2006, seminal work established that retinal dissociates from postnatal day 4–6 mice were the optimal donor cell age for transplantation [3, 4], leading to the adoption of this donor cell age for many subsequent murine to murine photoreceptor transplantations [5–10].

Many groups have since reported that transplantation of post-mitotic donor photoreceptors resulted in visual recovery, and these functional improvements were thought to be correlated to the number of donor cells migrating and integrating into the mouse ONL [6–8]. However, later studies demonstrated that transplanted mouse photoreceptors rarely integrate with the recipient retina and, instead, transfer cytoplasmic contents, such as the fluorescent GFP tag used to label the donor cells, to the host retina, in a process termed material transfer [11–15]. Further investigations into this phenomenon have demonstrated that transfer is bidirectional (donor to host and host to donor), requires direct contact, and is mediated through the formation of nanotube-like protrusions between photoreceptor cells [11–17].

Though cell integration may not the main mechanism for visual recovery, cell transplantation to the retina still holds promise. Material transfer has the potential to be leveraged as a therapy to deliver organelles or proteins involved in visual transduction from healthy donor cells to rescue diseased photoreceptors. Mouse photoreceptors have been shown to transfer RNA, cytoplasmic proteins, and organelles; however, for this to be a feasible strategy, it is important to determine if human photoreceptors can engage in material transfer.

The majority of reported studies on human photoreceptor transplants have used mouse models of end-stage retinal degeneration [18–23], with no (or very few) remaining host photoreceptors, thus obviating material transfer and consequently, making it difficult to investigate. In 2009, Lamba et al. [24] transplanted retinal cultures derived from human embryonic stem cells (hESCs) that had undergone a 3 week retinal determination period in adherent culture using IGF-1, noggin, and Dkk1. The donor cells were labelled with a cytoplasmic GFP reporter under ubiquitous promoters (hEF1α and CMV) and were transplanted into wildtype and Crx^−/−^ mice. GFP^+^ cells were detected in the ONL 3 weeks post-transplantation, though now with the new insights of material transfer, it is unclear if these cells have truly integrated.

Differentiation of human photoreceptors from embryonic and induced pluripotent stem cells have evolved since these first retinal determination protocols, which were dependent on exogenous factors to target BMP and Wnt signaling [25]. Protocols to generate retinal organoids, which are self-forming three dimensional optic cups, provide additional intrinsic signalling pathways to produce more physiologically relevant photoreceptors with mature and functional outer segments [26–33]. The creation of photoreceptor-specific reporter lines or viral-mediated cell labelling have provided the field with tools to purify and track photoreceptors in vivo [19, 34–36]. We wondered if human photoreceptors derived from these newer protocols have the capacity to transfer material and if this is stage specific.

We transplanted photoreceptors isolated from human stem-cell derived retinal organoids from different ontogenetic stages into both immune suppressed *Nrl*^*−/−*^ mice and immune deficient *NSG* mice to look at transfer of human-specific cargo. As cytoplasmic cargo transfers more readily than nuclear proteins, we confirmed the identity of our donor cells through the endogenous nuclear CRX-GFP reporter and used human specific antibodies to track cargo that may transfer to the mouse ONL, such as mitochondria and recoverin. Regardless of donor cell maturity or recipient background, we did not detect any transfer of human cargo to the mouse retina; instead, there were rare instances of integration.

To rule out the effect of a species differences between the human donor cells and recipient mouse retina, we then used in vitro cultures to investigate material transfer between human photoreceptors dissociated from human retinal organoids with cytoplasmic photoreceptor-specific reporters. While fluorescent proteins were able to transfer in the human-to-human co-cultures, those of mixed human photoreceptors and mouse retinal dissociates did not. The more dispersed distribution between mouse and human photoreceptors in culture compared to human photoreceptor cultures alone suggest that the lack of contact between the two species (human-mouse) in vitro prevents transfer. This study is the first to show that human stem-cell derived photoreceptors have the capacity to transfer material, which could be leveraged in the future to develop delivery strategies to treat retinal degeneration.

## Methods

###  Stem cell culture and maintenance

H9, CRX-GFP H9 (gift from Dr. Majlinda Lako, NewCastle University, United Kingdom), and Nrl-eGFP H9 and CRX-tdTomato H9 (gift from Dr. David Gamm, University of Wisconsin-Madison, WI, United States of America) cell lines were maintained on Geltrex coated plates in Essential 8 (Thermofisher Scientific, Mississauga, ON, Canada), or mTESR^+^ (STEMCELL Technologies, Vancouver, ON, Canada) medium and passaged at 75–80% confluence using ReLeSR (STEMCELL Technologies, Vancouver, BC, Canada). Routine mycoplasma testing was performed using the MycoAlert Mycoplasma Detection kit (Lonza, Basel, Switzerland).

### Retinal organoid differentiation

Retinal organoids were differentiated according to previously established protocols, with some modifications [32, 33, 37, 38]. Media formulations are listed in Additional file 1: Table S1. After reaching 90–95% confluence in Essential 8, Essential 6 (Thermofisher Scientific, Mississauga, ON, Canada) was used to initiate the differentiation period for 2 days. The cells were then fed with proneural induction medium (pNIM) for 3 weeks, during which laminated optic vesicle structures would appear. A P200 pipette tip was used to scrape an approximate 4 mm grid pattern before using a cell scraper to detach the retinal organoids after 21–28 days. The detached organoids were allowed to settle to the bottom of the falcon tube by gravity (~ 3–5 min) and washed 3 times in DMEM to remove single cells and debris. The organoids were then cultured in retinal initiation media (RIM) in poly-HEMA (Sigma Aldrich, Mississauga, ON, Canada) coated plates. The organoids were separated from clumps or nonretinal tissues using 2 sterile needles. Organoids were denoted as 4 weeks old after transitioning to suspension culture. Retinal maturation media (RMM) was supplemented with 10% FBS and taurine at week 6 (RMM-RA), with retinoic acid (1 μM) added to the media at week 10 (RMM + RA). The concentration of retinoic acid was reduced to 0.5 μM at week 12 and the media was further supplemented with 1% N-2 supplement (Final Retinal Maturation Media). Media changes were performed 2–3 times per week.

### Immunohistochemistry

Organoids were fixed in 4% paraformaldehyde (PFA) for 20 min, washed in PBS, and immersed in 30% sucrose overnight. Samples were embedded in OCT and frozen in isopentane cooled with dry ice. Cryosections of 20 μm were collected for immunohistochemistry. Sections were permeabilized in PBS with 0.3% Triton-X for 15 min and blocked in 10% donkey serum diluted in PBS with 0.1% Triton-X for 1 h at room temperature. Primary antibodies (listed in Additional file 1: Table S2) were diluted in 1% donkey serum with 0.1% Triton-X overnight at 4 °C. Slides were washed with PBS + 0.1% Triton-X (PBST) 3 times to remove unbound primary antibody. Secondary antibodies were diluted in 1% donkey serum + 0.1% Triton-X in PBS and incubated for 1 h at room temperature in the dark. The sections were washed 3 times in PBST, counter-stained with Hoechst 3342 at 1:1000 dilution in PBS (Cell Signaling Technology, Danvers, MA, USA) for 11 min, washed an additional three times in PBS, and mounted with ProLong Gold Antifade (Thermofisher Scientific, Mississauga, ON, Canada).

### RNA extraction, cDNA synthesis, and qRT-PCR

Organoids were washed once in PBS and lysed in RA1 buffer from the Nucleospin RNA II Kit (Macherey–Nagel, Düren, Nordrhein-Westfalen, Germany) according to the manufacturer’s instructions. Samples were either frozen at − 80 °C immediately or processed for RNA extraction. Lysates were sonicated for 3 min at room temperature and then further processed according to kit instructions. An additional DNase removal step was performed for 15 min at RT. Purified RNA was stored at − 80 °C. For cDNA synthesis, 250 ng of RNA was used per 20 μl reaction using the Superscript VILO synthesis cDNA kit (Invitrogen, Waltham, Massachusetts, USA). RNA used for cDNA synthesis had minimum 260/280 values ranging from 1.8 to 2.0.

For 10 μl qRT-PCR reactions using Sso Advanced SYBR Green (Bio-Rad Laboratories, Hercules, California, USA), 2 μl of cDNA (diluted 1:5) was added to 1 μM of forward and reverse primer mix. qRT-PCR was performed using the QuantStudio 6 Flex Real Time-PCR System (Thermofisher Scientific, Mississauga, ON, Canada) for 40 cycles using an annealing temperature of 55 °C. Primers sequences (Additional file 1: Table S3) were optimized in silico and validated using a 4-point standard curve to ensure that efficiencies were between 85 and 115%. Primer specificity was determined by the presence of a single melt curve or single PCR product resolved on an agarose gel. Cycle threshold (Ct) values were normalized to GAPDH as a reference gene (ΔCt) and normalized undifferentiated H9 cells as a reference sample (ΔΔCt). Fold change was calculated by the 2^−(ΔΔCt)^ method.

### Organoid dissociation

Organoids were dissociated using the Worthington Papain Dissociation Kit (Worthington Industries, Columbus, OH, USA) as per the manufacturer’s instructions. After 40–50 min of incubation in papain, organoids were triturated into a single cell suspension and neutralized with media. Cells were pelleted at 300*g* for 10 min and resuspended in a solution of Earle’s Balanced Salt Solution with ovomucoid inhibitor and DNase I. The cell suspension was gently layered on top of 5 ml of ovomucoid inhibitor, and the ovomucoid gradient was centrifuged at 60*g* for 10 min, with the acceleration/deceleration switched off, to remove debris and dead cells. The cell pellet was resuspended in PBS and passed through a 40 μm cell strainer. Viability and cell counts were assessed by trypan blue. For mouse in vitro cultures, retinas were dissected from postnatal day 3–5 mice in cold CO_2_-independent media and dissociated using the Worthington Papain Dissociation Kit using the same protocol.

### Cell sorting

Organoids were dissociated as described above. Cells were resuspended into FACS buffer (2% BSA, 25 mM HEPES, 300 U/ml DNase I in PBS) and strained through a 40 μm cell strainer. 7-AAD (1:50 dilution) was used for live/dead discrimination. After gating for single live cells on the BD Aria III, Aria IIIu, and Melody, photoreceptors were gated using GFP. A blank BV421 or APC channel was used to gate out weak auto-fluorescent cells. Cells were sorted into cold collection buffer consisting of 50% DMEM and 50% FBS. After FACS, cells were pooled, washed with PBS, and centrifuged at 300*g* for 15 min. Viability and cell counts were confirmed using trypan blue and a hemocytometer prior to cell transplantation.

### Cell transplantation

Animal work was approved by the University Health Network Animal Care Committee (protocol 3499), the Canadian Council on Animal Care guidelines and the guidelines set by the Association for Research in Vision and Ophthalmology (ARVO). All animal experiments adhered to the ARRIVE guidelines. Male and female *Nrl*^*−/−*^*, C57BL/6J*, and *NSG* mice (6–16 weeks) were used as transplant recipients. Immune competent recipient animals, *Nrl*^*−/−*^ and *C57BL/6J*, (Additional file 1: Table S4) were given an intraperitoneal (IP) injection of cyclosporine at 30 μg/g bodyweight diluted in sterile 0.9% NaCl (Bioshop, Burlington, ON, Canada) for immunosuppression every day for 2 days prior to the transplantation day and continuing for 7 days more, after which cyclosporine added to the drinking water for the remainder of the study.

Animals were anesthetized by an IP injection of 50 mg/kg ketamine (Ketalean, 8KET004D, Bimeda MTC Animal Health Inc. Cambridge, ON, Canada) and 1 mg/kg medetomidine (Cepetor, 236 1506 0, Modern Veterinary Therapeutics LLC, Miami, FL, USA) prepared in sterile 0.9% NaCl. The pupils were dilated with 1% tropicamide (Mydriacyl, 0065-0355-03 Alcon, Mississauga, ON, Canada) and eyes were kept lubricated with Systane Gel (Alcon). The eye was immobilized with a custom latex dam and a plastic coverslip was used to view the fundus under a microscope. A sharp 30 G needle was used to make an incision in the sclera to allow for entry with a 33 G blunt needle. Once the blunt needle was positioned correctly in the subretinal space, a puncture in the cornea was made to relieve intraocular pressure. A total of 1 μl was delivered to the subretinal space (1. 75 × 10^5^ cells/eye) and the needle remained in position for an additional 30 s post-delivery to reduce the chance of cell reflux. For recovery, animals were returned to their cages that were warmed on a heating pad and given an IP injection of atipamezole of 1 mg/kg (Revertor, 236 1504 0, Modern Veterinary Therapeutics LLC, Miami, FL, USA).

Immune competent animals (*Nrl*^*−/−*^ and *C57BL/6J*) received a 1 μl intravitreal injection of triamcinolone acetonide (40 mg/ml) after cell delivery. Systemic cyclosporine injections (IP) were administered daily for 7 days after transplantation (30 μg/g bodyweight), followed by oral cyclosporine administered at 200 μg/ml in the drinking water for the remainder of the study, which was replaced weekly until tissue harvest (Neoral, Novartis, Cambridge, MA, United States).

### Tissue harvest

Animals were euthanized with an overdose of sodium pentobarbital and perfused transcardially with PBS and then 4% PFA. After enucleation and corneal puncture, eyes were further post-fixed in 4% PFA for 2 h at RT, washed 3 times in PBS, and left in 30% sucrose dissolved in PBS at 4 °C overnight. The eyes were equilibrated in a 1:1 solution of 30% sucrose: OCT for 1 h before embedding. OCT blocks were flash frozen and stored at − 80 °C until sectioning. Retinal sections were cut at 20 μm thickness and processed with the same immunohistochemistry protocol as stated above. Quantifications of GFP^+^ donor cells were performed in serial sections, where GFP^ +^ donor cells in the subretinal space were quantified in every 5th slide. Integration was calculated by normalizing the number of GFP^+^ cells that were quantified in the ONL with the number of GFP^ +^ cells in the SRS. Transplant recipients with less than 50 donor cells in the SRS were excluded from analysis.

### In vitro co-culture

Human retinal organoids (Nrl-eGFP H9 and CRX-tdTomato H9) and retinas isolated from postnatal day 3 to 5 mice (*Nrl::GFP*) were dissociated as described earlier using papain and resuspended at 1 × 10^6^ cells/ml in final retinal maturation media. For co-culture, 1 × 10^5^ cells/well of each population were seeded into a 96-well plate. Media changes were performed every 3 days on the in vitro retinal dissociates.

To harvest the cells for flow cytometry, papain was used to lift the cells (40–50 min at 37 °C with trituration). Media was added to neutralize the dissociation and the cells were then centrifuged at 300*g* for 10 min in a 96-well V-bottom plate to pellet the cells. After washing with flow staining buffer (0.5% BSA, 0.05% sodium azide in PBS), UV-Zombie fixable viability dye (1:800, diluted in PBS) was used to stain dead cells at room temperature for 10 min. The cells were washed twice, resuspended in flow buffer, and kept in the dark until ready for analysis. The samples were analyzed on the day of harvest at each time point using *Nrl::GFP*, Nrl-eGFP H9 and CRX-tdTomato H9 cells that were cultured separately and mixed 1:1 right before data acquisition as a baseline control.

### Mitotracker staining and co-culture

To stain for Mitotracker, cells were incubated in 100 nM MitoTracker™ Red (Thermofisher Scientific), diluted in DMEM, at 37 °C for 30 min, after which the cells were pelleted by centrifuging at 300*g* for 15 min. The stained cells were washed in PBS and spun down at 300*g* for 15 min, 3 times. For the final wash, the cells were washed in DMEM and after centrifugation, the supernatant was collected to add to unstained cells as a control to ensure that there was no free MTR dye that could incorporate into the cells during the in vitro culture (known as the wash control). The cells were harvested on day 3 and stained with UV-zombie fixable viability dye. After staining, the samples were washed in FACS buffer twice and fixed in 4% PFA at room temperature, for 10 min. The cells were washed 3 times in FACS buffer and stored at 4 °C in the dark until flow acquisition.

### Flow cytometric analysis

Data was acquired on the BD LSR Fortessa X-20 and analyzed using FlowJo 10.7.1. The cell population of interest was identified through forward area (FSC-A) and side area (SSC-A) scatter. Doublets were excluded by forward scatter height (FSC-H) and forward scatter width (FSC-W) followed by side scatter height (SSC-H) vs side scatter width (SSC-W). Live cells were gated by the UV-zombie fixable viability dye, and biaxial plots were generated with MTR versus CRX-GFP. For cocultures with photoreceptor-specific fluorescent reporters, one recipient population reporter line (Nrl-eGFP^+^) was gated first from the bulk population of live cells, followed by the donor population reporter (tdTomato^+^) to quantify the number of double positive photoreceptors. Gating was set using the FMO (fluorescence-minus one) controls and the day 0 stained samples to establish baseline fluorescence.

### Imaging

Images were acquired on an Olympus FV1000, Zeiss LSM 780 or 880 point scanning confocal microscope using 20X air, 40X air and oil, and 63X oil immersion lenses. For quantification of donor cells after transplantation, whole sections were scanned on the Axioscan slide scanner. Live imaging of dissociated retinal organoids was performed on the Zeiss AxioObserver Z1 spinning disk confocal equipped with an incubator to maintain a constant temperature of 37 °C, 5% CO_2_, and humidity.

### Statistical analysis

Data is presented as mean ± SEM. Statistical tests were performed in Graphpad Prism 9.1.1. For comparisons made between two groups, a *t* test was performed. For a comparison made between two or more groups, a one-way ANOVA was performed with Tukey’s post-hoc. Comparisons with more than one independent variable were analyzed using a two-way ANOVA with Sidak’s post-hoc.

## Results

### Differentiation and isolation of human photoreceptors from stem-cell derived retinal organoids

**Fig. 1:**
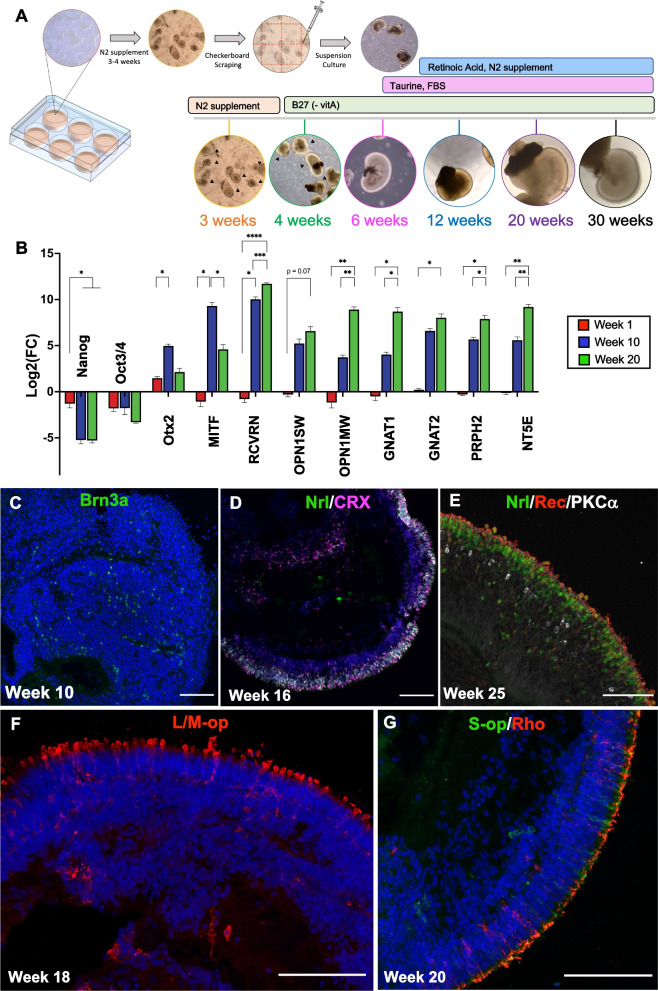
Generation of retinal organoids from human pluripotent stem cells. **A** Schematic showing the retinal organoid differentiation protocol. Human pluripotent stem cells (H9) were grown to confluence and pushed towards a neuroectoderm lineage with a 3–4 week induction period. Laminated structures (denoted in black triangles in the 3–4 week brightfield insets) were excised and matured in suspension culture. **B** Gene expression of H9-derived retinal organoids. Fold change was calculated by the ΔΔCt method, with GAPDH as the house keeping gene and normalized to undifferentiated H9 cells. Fold change is presented as mean ± SEM (n = 3 biological replicates using 7–10 pooled organoids per sample), analyzed by one way ANOVA with Tukey’s post-hoc. *p < 0.05; **p < 0.01; ***p < 0.001; ****p < 0.0001. **C** Brn3a^+^ retinal ganglion cells (green) in a week 10 retinal organoid. **D** Week 16 retinal organoid expressing CRX (pink) and NRL (green). **E** Week 25 retinal organoids expressing recoverin (red), NRL (green), and PKCα white.** F** Mature L/M cone photoreceptors (red) at week 18 and **G** S-cone photoreceptors (green) and rod photoreceptors (red) at week 20. Scale bar = 100 μm

We differentiated retinal organoids from human embryonic stem cells using a modified approach from previously published 2D confluent to 3D protocols [32, 33] and harvested the laminated optic-like vesicle by scraping the well, as first reported by Cowan et al. [38] and Regent et al. [37]. Overgrowing the stem cells to confluence induced spontaneous differentiation and generation of laminated tissues, or retinal organoids, which appeared after 3 weeks in an N-2 supplemented neural differentiation media (Fig. 1A). We first dissected the laminated structures and accompanying retinal pigmented epithelial (RPE) cells manually, but later adopted bulk scraping of the well to improve the efficiency and yield. We observed that scraping the well in a 4 mm grid pattern before lifting with a cell scraper helped break up the cell sheets and reduce clumping, allowing the generated neural retinal tissues to be maintained in suspension culture. Harvesting the retinal organoids from the 2D plate should occur between 3 and 4 weeks, as the laminated structures begin to bud off and are gradually lost after this window. As the retinal organoids matured in suspension culture, we periodically pruned the cultures to remove non-laminated tissues and to prevent organoids from merging together.

Quantitative RT-PCR from bulk lysed organoids was used to benchmark the developmental trajectory of retinal organoids cultured with our protocol modifications relative to previous reported studies and in vivo development. Gene expression analysis of pooled retinal organoids show a significant increase in genes associated with photoreceptor development over time, such as *OTX2* and *RCVRN*; genes specific to cone photoreceptors *OPN1L/MW* and *GNAT2*; *GNAT1* in rod photoreceptors and *PRPH2*, an outer segment marker (Fig. 1B). Immunohistochemistry was used for spatial characterization of the retinal organoids. The forebrain and photoreceptor progenitor marker Otx2 and pan-photoreceptor marker recoverin were observed as early as week 6, though this was much more appreciable from week 8 onward (Additional file 1: Figure S1A). Brn3a^+^ ganglion cells were detected in the retinal organoids at week 10 (Fig. 1C), but were lost as the organoids matured, likely due to the lack of vascularization and death of inner cell types, which has also been reported by others [30]. Photoreceptor progenitor cells in the outer rim of the organoid formed a thin neuroepithelial cell layer (Fig. 1D), with a second cell layer below forming the inner nuclear layer, identified by Chx10 (Additional file 1: Figure S1B) and PKCα staining (Fig. 1E). Rod and cone photoreceptors, identified by rhodopsin, S-opsin and L/M opsin, were present around differentiation weeks 18–20 (Fig. 1F, G). Consistent with other protocols [28, 31–33] and with a developmental trajectory similar to retinal development in vivo, our modified differentiation protocol gives rise to retinal cell types found in all three layers of the retina.

**Fig. 2:**
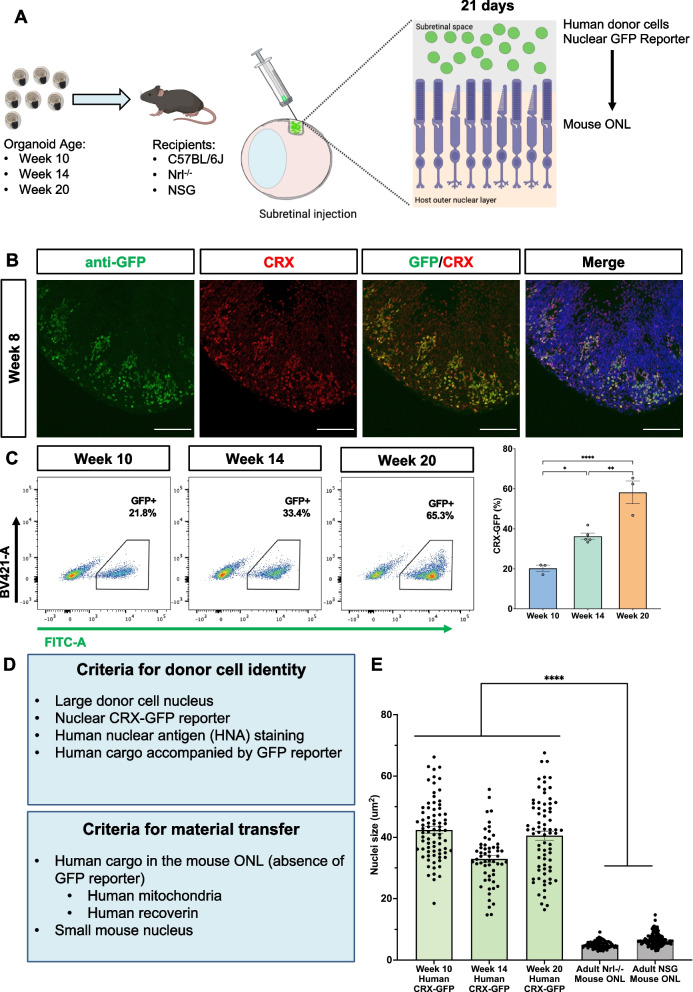
Transplantation of human photoreceptors to investigate material transfer. **A** Retinal organoids (derived from the nuclear CRX-GFP H9 reporter cell line) were dissociated at week 10, 14, and 20, FAC-sorted to isolate CRX-GFP^+^ photoreceptors and transplanted into adult *C57BL/6J*, *Nrl*^*−/−*^ or *NSG* mice. Tissue was collected 21 days later to look for evidence of material transfer. **B** Representative section of a week 8 retinal organoid with detectable GFP reporter (green) co-localized with CRX (red). Scale bar = 100 μm. **C** Quantification of CRX-GFP photoreceptor population in retinal organoids dissociated and sorted at weeks 10, 14, and 20. Live photoreceptors were gated for GFP (FITC) on the x-axis with an empty channel on the y-axis (BV421) to gate out weak auto-fluorescent cells. Data presented as mean ± SEM, analyzed by one way ANOVA with Tukey’s post-hoc. *p < 0.05; **p < 0.01; ****p < 0.0001 Sorts were performed on the Aria IIIu, BD Influx, and Aria III. **D** Criteria for donor cell identity and material transfer. **E** Donor human photoreceptors are distinguished by their significantly larger nuclei. Data presented as mean ± SEM (n = 57–114 nuclei from at least 3 animals in each group), one-way ANOVA with Tukey’s post-hoc. ****p < 0.0001

To isolate photoreceptors for our cell transplantation experiments (Fig. 2A), we generated retinal organoids from an H9 stem cell line with a nuclear CRX-GFP H9 reporter to ensure that the differentiation protocol proceeded as characterized earlier [34]. We were able to detect the presence of the GFP reporter as early as week 8 by IHC (Fig. 2B). We quantified the yield of CRX-GFP^+^ photoreceptors over time, which consisted of 20–65% of the overall retinal organoid population, in early (week 10), mid- (week 14), and late-stage (week 20) retinal organoids (Fig. 2C).

To investigate the relationship between donor cell maturity and material transfer we transplanted 1.75 × 10^5^ FAC-sorted CRX-GFP^+^ donor photoreceptors from early, mid- and late-stage retinal organoids into the subretinal space of either *C57BL/6J*, *Nrl*^*−/−*^ or immune deficient *NOD.Cg-Prkdc*^*scid*^* Il2rg*^*tm1Wjl*^*/SzJ* (NOD-SCID or *NSG*) mice. Eyes were harvested 21 days later, as mouse photoreceptor material transfer has been shown to peak at this time [16]. Due to the inefficient transfer of nuclear proteins compared to cytoplasmic proteins, we could not rely on tracking transfer of the nuclear CRX-GFP reporter. Instead, we investigated the transfer of human proteins into the mouse retina using human-specific antibodies (Additional file 1: Figure S2). Pilot data of week 14 photoreceptors transplanted into adult *C57BL/6J* mice showed survival of GFP^+^ donor cells in the subretinal space, and co-localization of nuclear GFP and human nuclear antigen (HNA) in donor cells in the subretinal space (Additional file 1: Figure S3). The transplanted human photoreceptor cells (30–40 μm^2^) are significantly larger than those of the mouse (5 μm^2^), which allowed us to discriminate between donor and host cells through nuclear morphology (Fig. 2D, E).

**Fig. 3:**
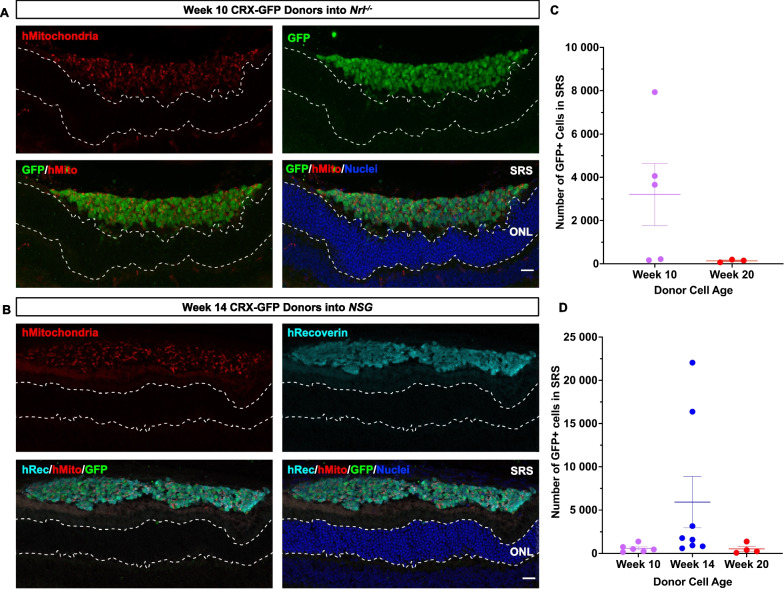
Human donor photoreceptors do not transfer cargo to the mouse ONL 21 days post-transplantation. **A** Representative image of week 10 CRX-GFP^+^ photoreceptors transplanted into *Nrl*^*−/−*^ recipients, stained for GFP (green), human mitochondria (red), and nuclei (blue). **B** Representative image of week 14 CRX-GFP^+^ photoreceptors transplanted into *NSG* recipients, stained for GFP (green), human recoverin (cyan), and human mitochondria (red). The dashed lines denote the host ONL. Scale bar = 20 μm. Quantification of donor cell number in the SRS of **C** *Nrl*^*−/−*^ and **D** NSG recipients 21 days post-transplantation, mean ± SEM (*Nrl*^*−/−*^ n = 3–5 per group, *NSG* n = 4–8 recipients per group). Four slides of serial sections were quantified per eye

We looked for the presence of cytoplasmic human cargo in the mouse ONL (in the absence of our nuclear human markers) as a measure of material transfer. Human mitochondria and human recoverin were co-localized with the GFP^+^ donor cell bolus in the subretinal space (Fig. 3). *Nrl*^*−/−*^ mice have been previously known to have a very high propensity for material transfer in mouse photoreceptor transplants [14], but we did not observe any detectable transfer between the different recipient backgrounds. The host ONL, below the donor cell bolus, was devoid of any detectable human mitochondria or human recoverin, which we hypothesized would transfer to mouse photoreceptors. Regardless of the maturity state of the donor cell population, we did not see markers for human mitochondria or human recoverin separate from cells expressing the nuclear GFP reporter, demonstrating that no cargo was transferred to the recipient host ONL 21 days after transplantation.

### Human photoreceptors migrate and incorporate into the mouse ONL 21 days post-transplantation

**Fig. 4:**
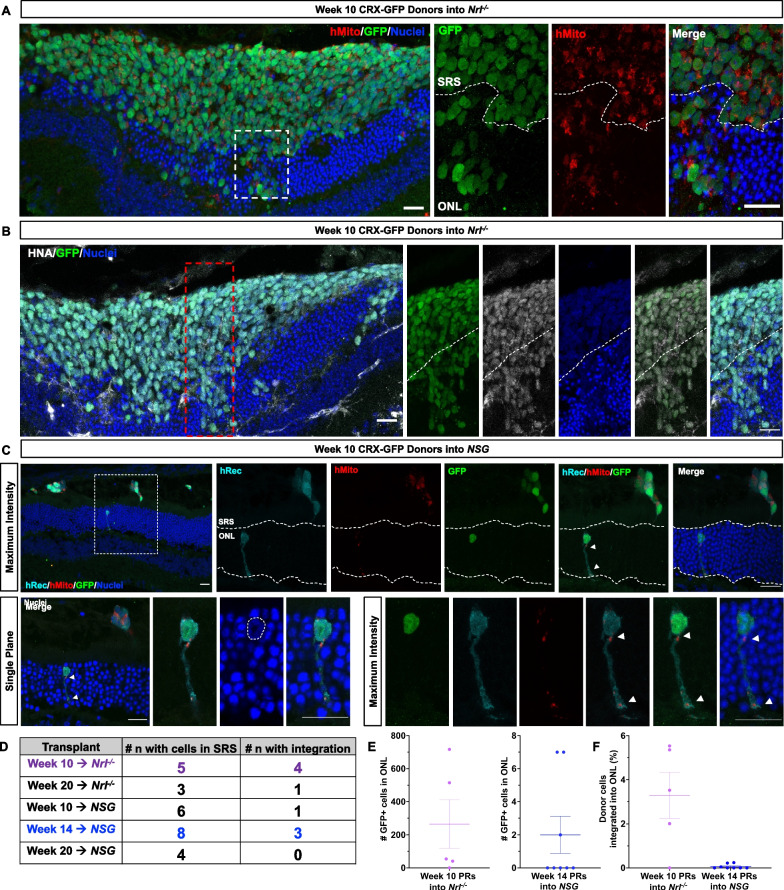
Transplanted human donor cells integrate into the recipient retina after 21 days. CRX-GFP^+^ photoreceptors were FAC-sorted from weeks 10, 14, or 20 retinal organoids and transplanted into the subretinal space of *Nrl*^*−/−*^ or *NSG* mice. **A** Week 10 CRX-GFP^+^/hMito^+^ donor photoreceptors migrate into the ONL of an *Nrl*^*−/−*^ recipient. Scale bar = 20 μm. **B** Donor cells localized to the ONL co-express human nuclear antigen (HNA). Scale bar = 20 μm. **C** Human mitochondria is co-localized with nuclear CRX-GFP and human recoverin inside the ONL of an *NSG* recipient. Higher magnification maximum intensity projection of week 10 CRX-GFP^+^ donor cell with hMito and hRecoverin located inside the ONL of an *NSG* recipient. Human mitochondria are localized within the donor cell neurite, co-localized with human recoverin. Single plane image showing a large human nucleus (CRX-GFP^+^) in the ONL is denoted by dotted white lines. Scale bar = 20 μm. **D** Table reporting the frequency of transplanted animals (n) with integration events. **E** Number of integrating GFP^+^ cells found in the outer nuclear layer for weeks 10 and 14 photoreceptor transplantations, mean ± SEM (n = 5–8 animals per group). **F** Percent donor cell integration normalized to the total donor cell number in the SRS. Mean ± SEM (n = 5–8 per group)

Unexpectedly, we identified a limited number of cells within the mouse outer nuclear layer (ONL) which stained positive for human mitochondria (Fig. 4A). Notably, these occurrences consistently coincided with the presence of the nuclear GFP^+^ reporter and human-sized nuclei, indicating that the donor cells migrated into the host ONL, as opposed to transferring material from donor to host. Additionally, the integrated GFP^+^ cells were found to be positive for HNA, further reinforcing cell integration and not material transfer (Fig. 4B). Human mitochondria in the mouse ONL was found to only co-localize within the cytoplasm or neurites of the integrated donor cells, as shown by the human recoverin staining accompanied with a large GFP^+^ human nucleus (Fig. 4C).

We observed integration in both *Nrl*^*−/−*^ and *NSG* recipient mice, with the three (early-, mid-, and late-) stages of donor cells that were transplanted (Fig. 4D). Less than 5% of week 10 donor photoreceptors (3.29 ± 1.05%) were found to be integrated in *Nrl*^*−/−*^ recipients, and less than 1% of week 14 donor photoreceptors (0.06 ± 0.04%) were integrated in *NSG* recipients (Fig. 4E, F). While a rare occurrence, we observed integration of week 10 human photoreceptors transplanted into NSG hosts and of week 20 photoreceptors transplanted into *Nrl*^*−/−*^ hosts (1 animal each) (Additional file 1: Figure S4). This observation underscores the infrequent nature of these integration events.

**Fig. 5:**
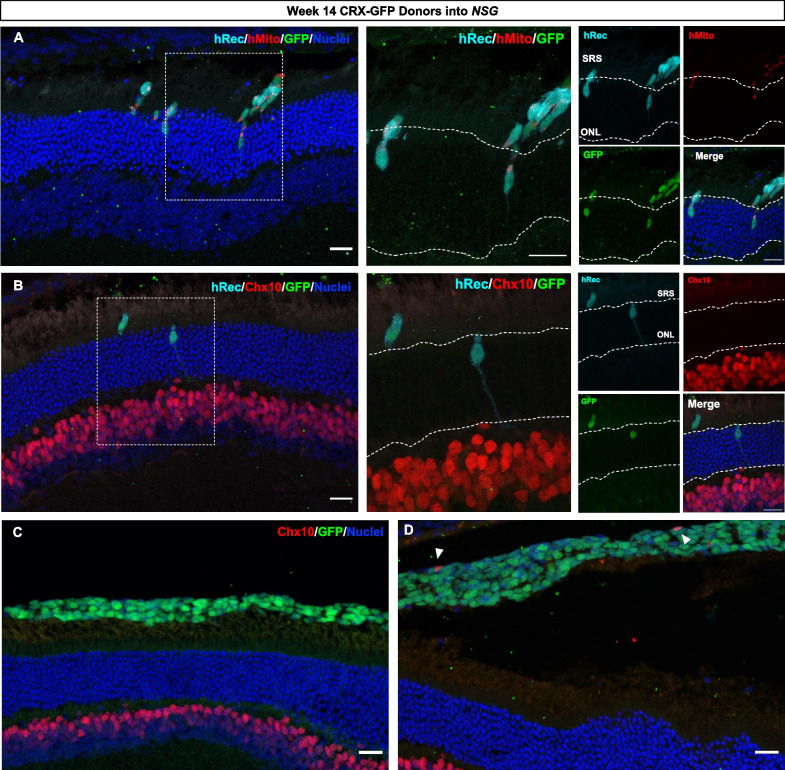
Transplanted human photoreceptors integrate into the *NSG* recipient retina after 21 days. **A** Week 14 CRX-GFP donor cell expressing human recoverin and human mitochondria located in the mouse ONL. **B** Migrating donor cells are Chx10^−^ and thus not bipolar cells. **C**, **D** Few GFP^+^ cells are Chx10^+^ (white triangles) in the donor cell bolus; however, none of these Chx10^+^ cells are observed in the ONL. Scale bar = 20 μm

Tracking our donor cells relies on CRX-GFP, a photoreceptor marker that is also expressed in bipolar cells [39], opening up the possibility that integrating GFP^+^ donor cells could potentially be a mixed population. To investigate this, we also stained with Chx10, which is a marker specific to mature bipolar cells. Chx10 expression was localized in the inner nuclear layer (INL) of the recipient mice, yet not among the integrating CRX-GFP^+^ donor cells, which stained for recoverin, thereby confirming that the transplanted cells were photoreceptors (Fig. 5A, B). While sporadic GFP^+^/Chx10^+^ cells were observed in the donor cell bolus in the SRS (Fig. 5C, D), these cells did not express human recoverin and are thus distinct from the cells that migrated into the host ONL. These observations demonstrate that our transplanted human photoreceptors did not transfer material to recipient mouse photoreceptors but did migrate and integrate into the mouse ONL.

### Human photoreceptors transfer cargo in vitro

**Fig. 6:**
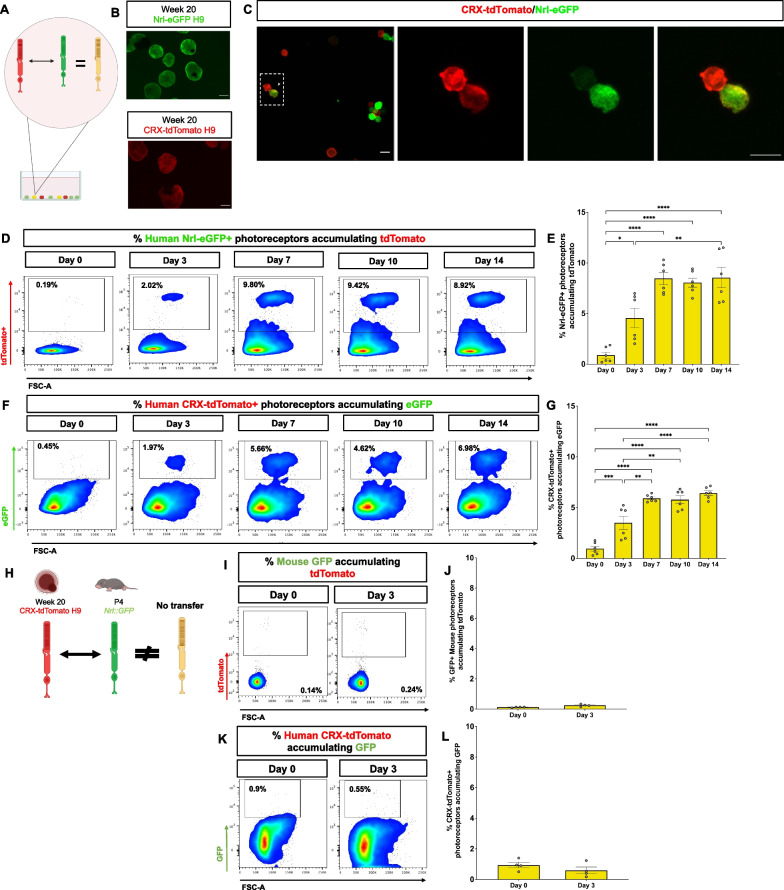
Human photoreceptors transfer cytoplasmic proteins in vitro but transfer between mouse and human photoreceptors is not detectable in vitro. **A** Schematic illustrating the human:human retinal organoid co-culture setup. Week 20 retinal organoids (CRX-tdTomato H9 and Nrl-eGFP H9) were dissociated and cultured 1:1 for 3, 7, 10, and 14 days. **B** Representative fluorescent images of CRX-tdTomato H9 and Nrl-eGFP H9 ROs at week 20. Scale bar = 500 μm. **C** On day 7, the co-cultures were dissociated and enriched for photoreceptors (eGFP^+^ or tdTomato^+^) by FACS. Collected cells were re-plated onto PDL/laminin coated dishes for immunohistochemistry. Representative image of a double positive (eGFP^+^/tdTomato^+^) photoreceptor. Scale bar = 10 μm. **D** Representative flow cytometry plots of live Nrl-eGFP^+^ photoreceptors gated for tdTomato over time. **E** Percentage of Nrl-eGFP^+^ photoreceptors that are accumulating tdTomato (double positive). **F** Representative flow cytometry plots of live CRX-tdTomato + photoreceptors gated for Nrl-eGFP. **G** Percentage of CRX-tdTomato photoreceptors accumulating eGFP (double positive). **H** Schematic illustrating the mouse:human retinal dissociate co-culture setup. Week 20 CRX-tdTomato H9 retinal organoids were co-cultured with retinal dissociates isolated from post-natal day 4 *Nrl::GFP* mice. **I** Representative flow cytometry plots of mouse rod photoreceptors (*Nrl::GFP*^+^) gated for tdTomato. **J** Quantification of mouse *Nrl::GFP*^+^ photoreceptors accumulating tdTomato. **K** Representative flow cytometry plots of human photoreceptors (CRX-tdTomato^+^) gated for GFP. **L** Quantification of human CRX-tdTomato photoreceptors accumulating GFP. Data are presented as mean ± SEM (n = 3–6) analyzed by one-way ANOVA with Tukey’s post-hoc. *p < 0.05; **p < 0.01; ***p < 0.001; ****p < 0.0001

Material transfer may be species specific, which could account for the lack of protein transfer observed from human to mouse photoreceptors in vivo. Another possibility is that human photoreceptors might lack the capacity for material transfer. To differentiate between these two possibilities, we utilized an in vitro assay to investigate whether material transfer takes place among human photoreceptors versus between human and mouse photoreceptors. We differentiated retinal organoids from the CRX-tdTomato and Nrl-eGFP H9 stem cell lines, which have cytoplasmic fluorescent reporters specific to photoreceptors, then dissociated and co-cultured the retinal organoid dissociates 1:1 for up to 14 days (Fig. 6A, B). We were able to detect double positive CRX-tdTomato^+^ and Nrl-eGFP^+^ cells by immunofluorescence (Fig. 6C, Additional file 1: Figure S5) after 7 days in vitro. To quantify transfer, we dissociated the retinal organoid co-cultures and analyzed them at each time point by flow cytometry. As an internal baseline for each timepoint, Nrl-eGFP or CRX-tdTomato retinal organoids were dissociated, cultured separately, and mixed before flow acquisition. The live Nrl-eGFP^+^ photoreceptor population was gated on tdTomato to quantify the percentage of cells accumulating red fluorescent protein (Fig. 6D, Additional file 1: Figure S6). There was a significant increase in the number of double positive photoreceptors over time, from less than 1% on day 0 to 8.5% on day 14 (Fig. 6E). Similarly, red cells acquired eGFP over time and up to 6.4% (Fig. 6F, G).

To investigate transfer of an additional cargo type in vitro, we dissociated and stained H9-derived retinal organoids with MitoTracker™ Red to label donor mitochondria and co-cultured with dissociated CRX-GFP^+^ retinal organoids as recipients (Additional file 1: Figure S7A). On day 3, there were 8.3 ± 0.6% of live cells double positive for MTR and GFP (Additional file 1: Figure S7B, C). These results suggest that dissociated CRX-GFP^+^ photoreceptors can also accept mitochondria, further demonstrating that human photoreceptors can transfer material.

### Mouse photoreceptors do not transfer material to dissociated human retinal organoids

After establishing that human photoreceptors are capable of transferring cytoplasmic proteins, we next asked if cytosolic proteins could be exchanged between human and mouse photoreceptors by co-culturing dissociated week 20 human retinal organoids derived from the CRX-tdTomato H9 cell line with retinal dissociates isolated from post-natal day 4 *Nrl::GFP* mice (Fig. 6H).

Mouse rod photoreceptors (*Nrl::GFP*^+^) comprised approximately 16% of the mouse:human co-culture on day 0 (Additional file 1: Figure S8). However, mouse photoreceptor survival in vitro is poor, declining to 1% of cells in the cultures by 7 days (Additional file 1: Figure S8). This poor survival has been reported before [40, 41] and occurs irrespective of whether the mouse cells are cultured alone or with human cells (Additional file 1: Figure S8). Thus, we only analyzed the co-cultures on day 3, which has been shown previously to be sufficient time to detect transfer [16]. There was no detectable transfer in the human:mouse co-culture when *Nrl::GFP*^+^ mouse photoreceptors were gated for tdTomato (Fig. 6I, J). Similarly, there were no tdTomato ^+^ human photoreceptors that had received GFP throughout the co-culture period (Fig. 6K, L), which demonstrates that there is little exchange of fluorescent proteins between human and mouse photoreceptors.

**Fig. 7:**
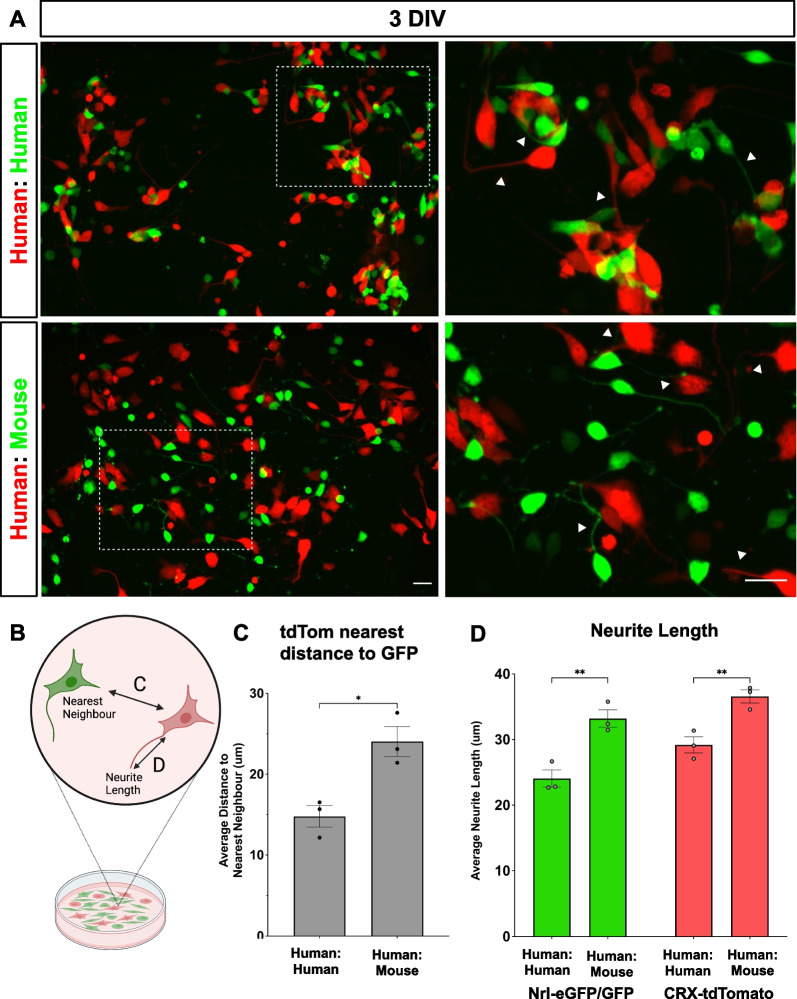
Dissociated human:human retinal organoid co-cultures cluster more closely together than human:mouse co-cultures. Week 20 retinal organoids and mouse retinal dissociates were cultured on PDL and laminin coated glass dishes for live imaging. Representative images after 3 DIV of **A** Human retinal organoid co-culture from CRX-tdTomato H9-derived retinal dissociates and Nrl-eGFP H9 derived retinal dissociates and *Nrl::GFP* mouse retinal dissociates co-cultured with human CRX-tdTomato H9 derived retinal organoid dissociates. Scale bar = 20 μm. **B** Schematic of quantifications for nearest neighbour and neurite length. **C** Nearest neighbour analysis was performed to quantify the average distance of the nearest neighbour between tdTomato and eGFP cells, analyzed by unpaired, two-tailed *t* test. *p < 0.05. **D** Average neurite length in human:human and human:mouse co-cultures. Data is presented as mean ± SEM (n = 3 biological replicates), analyzed by 2-way ANOVA with Sidak’s post-hoc test. **p < 0.01

Qualitative live imaging of human:human and human:mouse co-cultures showed that there were subtle differences in the distribution of the cell bodies. Red and green photoreceptors in the human: human co-cultures appeared to be clustered together compared to the mixed human:mouse co-cultures (Fig. 7A). To examine spatial differences and photoreceptor cell distribution between human:human and human:mouse co-cultures, we performed nearest neighbour analysis on day 3 (Fig. 7B, C). There was a significant decrease in the average distance to the nearest neighbour of CRX-tdTomato^+^ cells in in the human:human cultures (14.8 ± 1.3 μm distance to human Nrl-eGFP^+^ photoreceptors) versus human:mouse (24.0 ± 1.8 μm distance to mouse *Nrl::GFP*^+^ photoreceptors) (Fig. 7C). These results confirm that the cell bodies of the mouse cells are further apart from the human cells, suggesting that physical distance could be impeding transfer in vitro.

Average neurite length in the *Nrl::GFP*^+^ mouse photoreceptors (33.2 ± 1.3 μm) was significantly longer than Nrl-eGFP^+^ human photoreceptors (24.1 ± 1.3 μm) (Fig. 7D). Interestingly, the neurite length of the CRX-tdTomato^+^ human photoreceptors was also significantly different and dependent on co-culture with either human (29.1 ± 1.2 μm) or mouse (36.6 ± 1.0 μm) cells (Fig. 7D). Thus, the lack of detectable material transfer in between human:mouse photoreceptors likely stems from the greater distance between cell bodies. The closer clustering observed in the human:human co-cultures supports the idea that intercellular communication is restricted by species.

## Discussion

Material transfer between mouse photoreceptors has been well-characterized [11–17]; however, until this study, it was not clear whether human photoreceptors were capable of material transfer. We first investigated the possibility of human to mouse transfer in vivo, using recipient models with intact host photoreceptors, which then led us to corroborate our observations in vitro to test differences in material transfer due to species specificity.

Successful detection of material transfer is highly dependent on donor cell survival, which could be impacted by donor cell age and the host immune response after subretinal injection. Currently, there are no studies that report the optimal age for human photoreceptor transplants, as what MacLaren et al. [3] achieved when they first reported this for mouse photoreceptors. Human photoreceptors dissociated from week 12 (day 90) up to week 35 (day 250) retinal organoids have been transplanted into various mouse models of retinal degeneration; yet, differences in differentiation methods and rodent models makes it difficult to ascertain the optimal stage for cell survival [19, 20, 22, 33, 42, 43]. We also observed large variability in our donor cell survival regardless of cell maturity; however, the number of surviving GFP^+^ human donor cells in the subretinal space is still comparable to previously reported data with mouse photoreceptor transplants [16]. While mouse to mouse photoreceptor material transfer is well-documented [11–17, 44], we found no evidence of transfer of endogenous human protein and mitochondria to recipient mouse photoreceptors, in any of the models tested, 21 days post-transplantation, which is a time when transfer efficiency peaks in transplanted mouse photoreceptors [16]. Given the similarity of human and mouse donor cell survival in the subretinal space, it is unlikely that the lack detectable human to mouse material transfer is caused by low donor cell survival.

Interestingly, a small subset of transplanted human photoreceptors integrated into the mouse retina in lieu of transfer. Integration was observed in transplants with early, mid-range, and older donor cells and in both *Nrl*^*−/−*^ and *NSG* recipients. We observed a higher proportion and rate of donor cell integration in *Nrl*^*−/−*^ recipients, which may be due to the breakdown of the outer limiting membrane (OLM) compared to the wild type recipients [45]. Previous transplantation studies used DL-alpha-aminoadipic acid (AAA) to disrupt the OLM, which resulted in a greater number of GFP^+^ cells in the mouse ONL [46, 47]. Similarly, disruptions in the OLM in *Nrl*^*−/−*^ mice could facilitate human donor cell migration into the mouse ONL.

Other groups have observed human photoreceptor incorporation into the mouse retina after transplantation [33, 43]. Gasparini et al. [43] transplanted day 200 (week 28) cone photoreceptors into *Cpfl1* mice and saw minimal donor-host interaction at 3 weeks, with more extensive graft integration at 10- and 26-weeks post-transplantation. This may be attributed to degeneration and rosetting of the *Cpfl1* retina, similar to what we observed in the *Nrl*^*−/−*^ recipients [43]. This graft integration was distinct from material transfer, as there was no measurable transfer of cytoplasmic GFP from human to mouse photoreceptors [43], which is in agreement with our findings. Interestingly, transplants with an older donor cell population, day 250 cones (week 35), did not incorporate into the *Cpfl1* retina, which suggests that cell maturity could also play a role in cell integration [43]. We lack an understanding of why only a small proportion of human photoreceptors can migrate into an already established neuronal layer. It is possible that photoreceptors exhibit heterogeneity in their migration capabilities, which is a plausible hypothesis, considering that other retinal neurons exhibit multiple modes of migration [48]. As mechanisms mediating photoreceptor migration and translocation are not as well characterized compared to other retinal neurons, such as ganglion cells, it is possible that only a subset of photoreceptors have the capacity to migrate and integrate into the ONL [48, 49].

Transfer of fluorescent cargo was successfully detected by flow cytometry in cultures of dissociated human retinal organoids in vitro, which plateaued after 7 days. This plateau could be due to reaching a steady state, as material transfer is a function of both the rate at which labile cargo accumulates and its rate of decay. Ortin-Martinez et al. [16] demonstrated that while there were surviving donor cells in the subretinal space 90 days post-transplantation, the efficiency of GFP transfer in vivo was highest at 21 days. The use of non-labile cargo, such as tracking transfer of Cre recombinase, would be a more indicative readout of cumulative transfer.

Our data show that photoreceptors from dissociated retinal organoids transfer cytoplasmic material in vitro, suggesting that the lack of transfer in vivo is due to the species difference between donor and host. This was further corroborated by the lack of detectable transfer between human CRX-tdTomato photoreceptors and mouse *Nrl::GFP* rod photoreceptors in vitro and is consistent with results presented by Ortin-Martinez et al. [16], where they reported that transplanted human glioblastoma cells also did not transfer to the recipient mouse retina. This species specificity of material transfer could arise from variations in cargo handling, including the breakdown of xenogeneic material by recipient cells, thus preventing accumulation of detectable levels of transferred cargo. However, this scenario may not be likely as TNT-mediated mRNA transfer between human and mouse cells has been previously reported [50]. Alternatively, a requirement for species-specific intercellular interactions may preclude transfer between human and mouse cells. This possibility is supported by live imaging and nearest neighbour analysis, where we demonstrated that photoreceptors in human:mouse co-cultures are spatially separated compared to human:human photoreceptor cultures.

Though both human and mouse photoreceptors formed protrusions in co-culture, there were significant differences in the spatial distribution of the cell bodies and differences in neurite length, suggesting that a lack of cell interaction could be impeding transfer between the two species. Human:mouse photoreceptor interactions may be limited by inhibitory secreted factors or other non-photoreceptor cell-related mechanisms. Further research on investigating the interplay between spatial proximity, neurite outgrowth, nanotube formation, and physical contact in the context of material transfer will help guide future mechanistic analysis of material transfer. Due to the inherent limitation of culturing dissociated mouse retinal cells and their poor in vitro survival, it was not possible to extend our human:mouse coculture beyond three days; however, this timeline is still within the window in which mouse:mouse have been shown to transfer fluorescent proteins to other mouse photoreceptors in vitro [16].

The lack of detectable human to mouse photoreceptor transfer in vivo suggests that xenograft models are more appropriate systems to investigate cell replacement and engraftment therapies rather than material transfer. We demonstrated that human photoreceptors transferred material in vitro, though dissociation of both eGFP and tdTomato human retinal organoid populations abrogates the spatial orientation of the in vivo transplant environment, with the donor and host cells evenly dispersed across the dish and not densely packed as a bolus. Wagner et al. (2022, BioRxiv) developed an in vitro model of photoreceptor transplantation: FAC-sorted human photoreceptors were co-cultured atop whole organoid recipient for 6 weeks, after which they observed incorporation of the graft onto the host organoid [51]. GFP and RFP reporters were used to discriminate between donor and host photoreceptors, respectively, and while no overlap was observed, these reporter lines were nuclear, which do not transfer as readily as cytoplasmic reporters and therefore may not be as easily detectable [51]. In vitro models such as using an intact recipient retinal organoid may also be useful to investigate intercellular communication between human photoreceptors with cytoplasmic reporters.

## Conclusions

In summary, we found that human retinal organoid photoreceptors at three different stages of maturity showed no evidence of material transfer in the mouse retina. A small subset of transplanted human photoreceptors had the capacity to integrate into the outer nuclear layer. Importantly, we show, for the first time, that human photoreceptors are capable of material transfer material to other human photoreceptors, suggesting that this strategy may be leveraged for therapeutic benefit.

### Supplementary Information

**Additional file 1**. Supplemental tables and figures.

## Data Availability

Data will be made available upon request.

## Abbreviations

eGFP: Enhanced green fluorescent protein
GFP: Green fluorescent protein
FACS: Fluorescence activated cell sorting
FBS: Fetal bovine serum
hESC: Human embryonic stem cell
HNA: Human nuclear antigen
ONL: Outer nuclear layer
CRX: Cone rod homeobox
Nrl: Neural leucine zipper
SRS: Subretinal space
tdTomato: Tandem dimer Tomato
