# Supplementary material for: Transplanted human photoreceptors transfer cytoplasmic material but not to the recipient mouse retina
Source: Stem Cell Res Ther. 2024 Mar 14;15:79. doi: 10.1186/s13287-024-03679-3 (PMC10941468; doi:10.1186/s13287-024-03679-3)
Supplement: Supplementary file 1 — Additional file 1. Supplemental tables and figures. [file 13287_2024_3679_MOESM1_ESM.pdf]

Supplemental Material

Supplemental Table 1. Media Formulations for Retinal Organoid Differentiation

Proneural Induction Medium: Weeks 0-4

- Advanced DMEM/F12 (Gibco 12634010)
- 1% GlutaMax (Gibco 35050061)
- 1% N2 Supplement (Wisent 305-016-IL)
- 1% Pen-Strep (Wisent 450-201-EL)

Retinal Initiation Media: Weeks 4-5

- 3X DMEM + GlutaMax (Gibco 10569-010)
- 1X F12 +GlutaMax (Gibco 31765-035)
- 2% B27 Supplement (minus vit A) (Gibco12587001)
- 1% NEAA (Gibco #11140-050)
- 1% Pen-Strep

Retinal Maturation Media without RA: Weeks 6-9

- 1% NEAA
- 2% B27 Supplement without vit A
- 10% ES-qualified FBS (Wisent 920-040)
- 1% Pen-Strep
- 100 µM taurine (Sigma T0626)
- (3:1) DMEM+ GlutaMax and F12 + GlutaMax

Retinal Maturation Media with RA Weeks 10-12

- Per 100 ml media: add 1 µl retinoic acid, from 1 mM stock solution (Sigma R2625) to the Modified retinal maturation media (final concentration 1µM).

Final Retinal Maturation Media: Weeks 12+

- Add 1% N2 Supplement
- Reduce retinoic acid concentration to 0.5 µM.

Supplemental Table 2. Antibody Information

Antibody	Host	Dilution	Manufacturer	Catalog #
**Primary Antibodies**				
Otx2	Goat	1:100	R&D Systems	BAF-1979
Chx10	Goat	1:500	Santa Cruz	sc-21690
Brn3a	Goat	1:750	Santa Cruz	sc-31984
CRX Clone 4G11	Mouse	1:800	Abnova	H00001406-M02
Recoverin	Rabbit	1:1000	Millipore Sigma	AB5585
Recoverin (Human), clone IJ23	Rabbit	1:500-1000	Sigma Aldrich	ZRB1107
Nrl	Goat	1:100	R&D Systems	AF2945
S-Opsin	Goat	1:1000	Santa Cruz	sc-14363
L/M Opsin	Rabbit	1:500	Millipore Sigma	AB5405
Rhodopsin, clone 4D2	Mouse	1:1000	Sigma Aldrich	MABN15
PKCa	Mouse	1:200	Santa Cruz	sc-8393
GFP	Goat	1:500	Rockland Immunochemicals	600-101-215
GFP	Chicken	1:500	Aves Labs	AB2307313
RFP	Rabbit	1:500	Rockland Immunochemicals	600-401-379
Human mitochondria	Mouse	1:500	Abcam	ab92824
Human nuclear antigen, Clone 235-1	Mouse	1:400	Millipore Sigma	MAB1281
**Secondary Antibodies**				
AlexaFluor Donkey anti-goat 488	Donkey	1:1000	Invitrogen	A11055
AlexaFluor Donkey anti-goat 568	Donkey	1:1000	Invitrogen	A11057
AlexaFluor Donkey anti-rabbit 568	Donkey	1:1000	Invitrogen	A31571
AlexaFluor Donkey anti-rabbit 647	Donkey	1:1000	Invitrogen	A31573
AlexaFluor Donkey anti-mouse 568	Donkey	1:1000	Invitrogen	A10037
AlexaFluor Donkey anti-mouse 647	Donkey	1:1000	Invitrogen	A10042
**Dyes**				
Hoechst 33342	N/A	1:1000	Cell Signalling Technology	4082
Zombie UV Fixable Viability Kit	N/A	1:800	Biolegend	423108
MitoTracker Red FM	N/A	100 nM	Invitrogen	M22425

Supplemental Table 3. qRT-PCR Primers

Target	Primer Sequences
GAPDH	**F:** AGCAAGAGCACAAGAGGAAGAG<br>**R:** GAGCACAGGGTACTTTATTGATGG
Oct3/4	**F:** CTGTCTCCGTCACCACTCTG<br>**R:** TGTGTTCCCAATTCCTTCCTTAG
Nanog	**F:** AATGGTGTGACGCAGAAGGC<br>**R:** TGCACCAGGTCTGAGTGTTC
Otx2	**F:** ACCTTGAACTCCACCTCTGC<br>**R:** GCTTCTCTTCTCTGACTCTCTTTG
MITF	**F:** TTGTCCATCTGCCTCTGAGTAG<br>**R:** CCTATGTATGACCAGGTTGCTTG
RCVRN	**F:** AAGCGAGCCGAGAAGATCTG<br>**R:** TGAGTGGTAGGTGGAGGGAG
OPN1SW	**F:** GTCTTCGTCGCCAGCTGTAA<br>**R:** GTAACCAGACCTGCTACAGTGC
OPN1/LMW	**F:** GAAGTTCAAGAAGCTGCGCC<br>**R:** TCTCACATTGCCAAAGGGCT
GNAT1	**F:** CACGATGCCCAAGGAGATGT<br>**R:** GGTGGTTGCAGATGCTGTTG
GNAT2	**F:** AGATGTGCTCCGATCCAGAG<br>**R:** TCATCTTCCACCAGCACCAT
Peripherin	**F:** GAGAGCCATTTTGTGCCCAA<br>**R:** ACACAGATAGCCAGGTACGG
NT5E	**F:** TAAGTCGCTCTGCCTCCAAA<br>**R:**TGGTTCCACTCCCACGTTAA

Supplemental Table 4. Mouse strains used in this study

Mouse strain	Supplier/Reference	Genotyping
C57BL/6J	The Jackson Laboratory<br>RRID:IMSR_JAX:000664	
Nrl::GFP	[48]	F: AAGTCGTGCTGCTTCATGTG<br>R: TTTCACTGGCTTCTGAGTCC
NOD.Cg-Prkdc^{scid} Il2rg^{tm1Wjl}/SzJ (*NSG*)	The Jackson Laboratory<br>RRID:IMSR_JAX:005557	
Nrl$^{-/-}$	[49]	F: TGAATACAGGGACGACACCA<br>R:GTTCTAATTCCATCAGAAGCTGAC

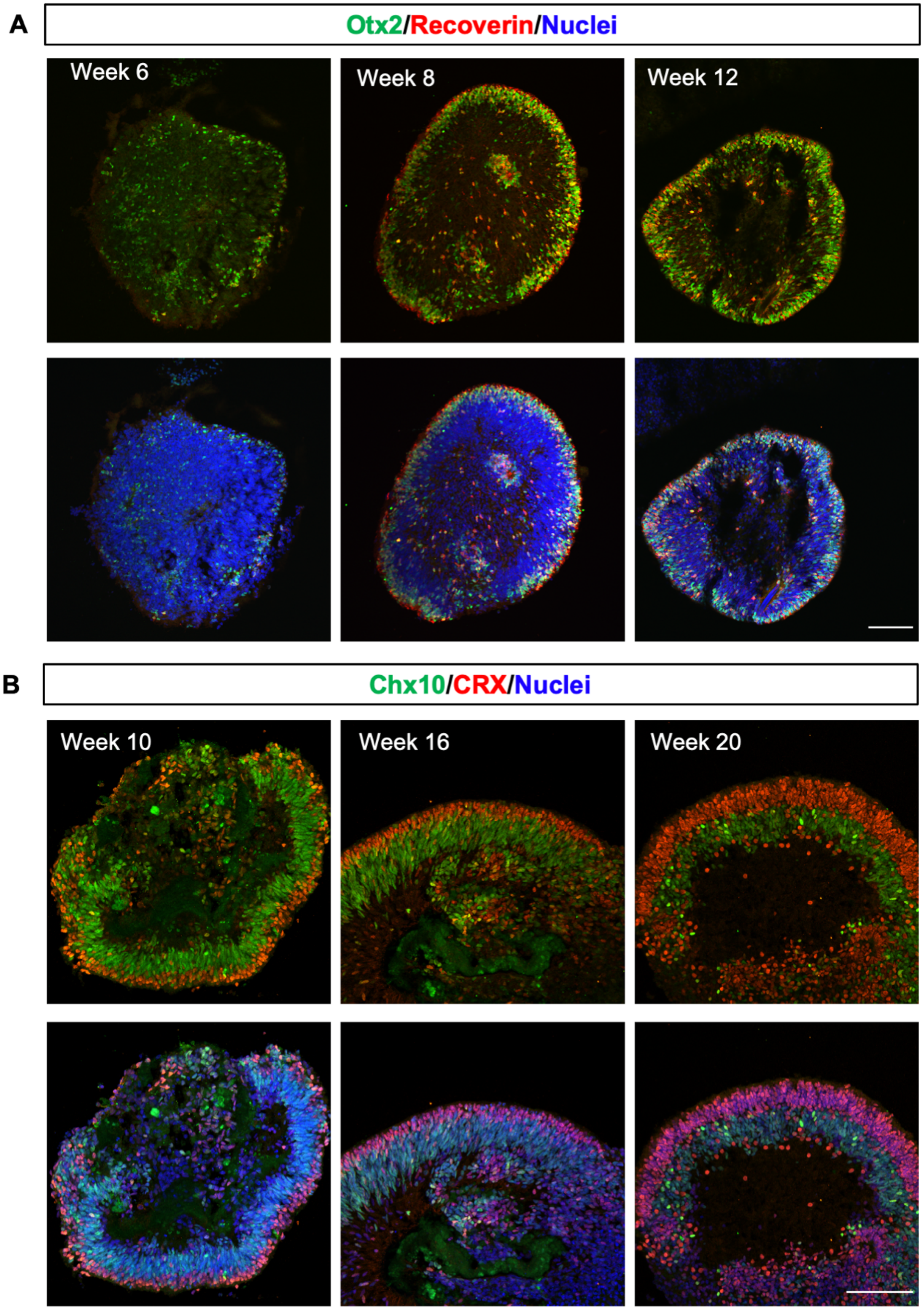

Supplemental Figure 1. Characterization of retinal organoids over time. A. Marker expression of photoreceptor precursors, Otx2 (green) and recoverin (red), from weeks 6 to 12. **B.** Marker expression of photoreceptors, shown by CRX (red) and bipolar cells, shown by Chx10 (green) from weeks 10 to 20. Scale bar = 100 μm.

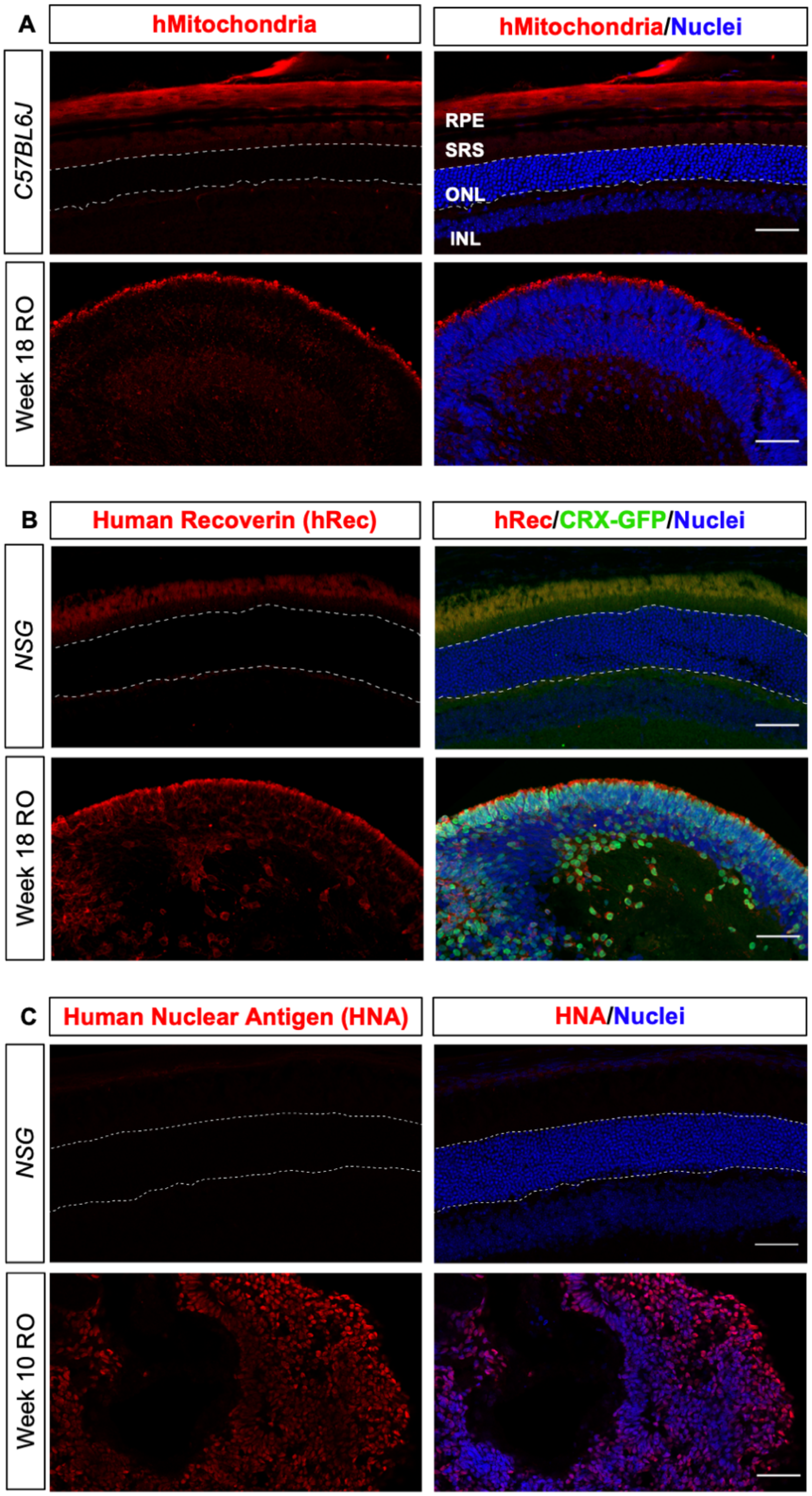

Supplemental Figure 2. Representative images of human-specific antibodies to identify donor photoreceptor cells that do not cross-react with mouse retinal tissue: **A**. human mitochondria, **B**. human recoverin, and **C**. human nuclear antigen. ONL: outer nuclear layer (demarcated between white dashed lines). INL: inner nuclear layer. SRS: subretinal space. RPE: retinal pigment epithelium. The human mitochondria antibody was raised in mouse and hence, has some background on the RPE of the mouse tissue. Scale bar = 50 μm.

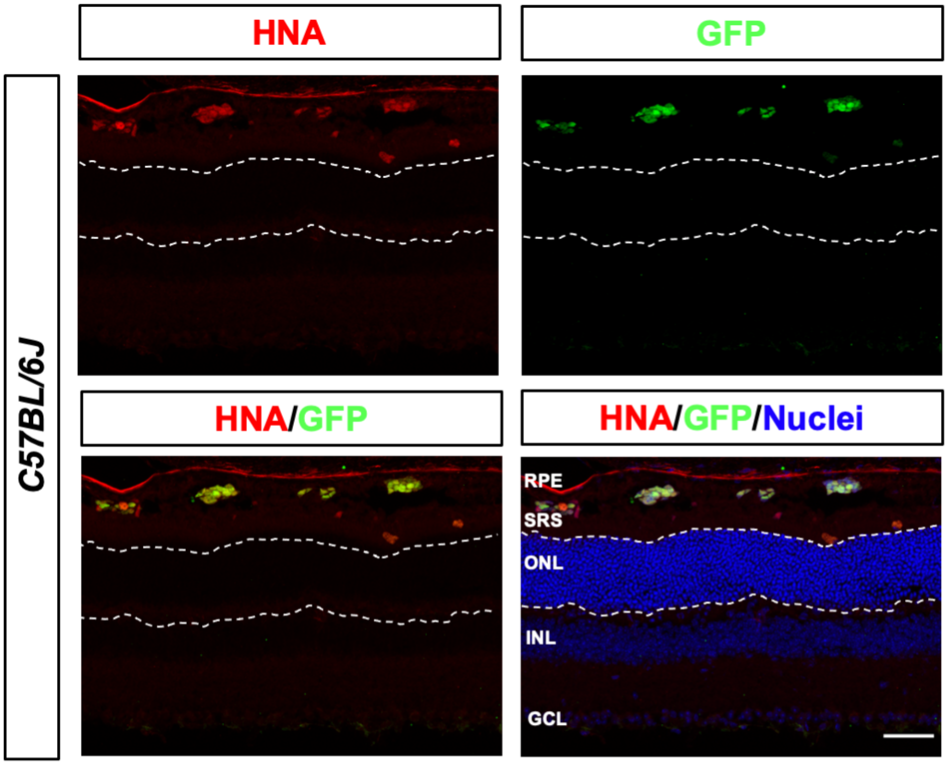

Supplemental Figure 3. Week 14 CRX-GFP+ photoreceptors survive in the subretinal space (SRS) 21 days after transplantation. Human nuclear antigen (HNA) co-localizes with the nuclear GFP reporter. The outer nuclear layer (ONL) is denoted between the 2 dashed lines. RPE: retinal pigment epithelium. INL: inner nuclear layer. GCL: ganglion cell layer. Scale bar = 50 µm.

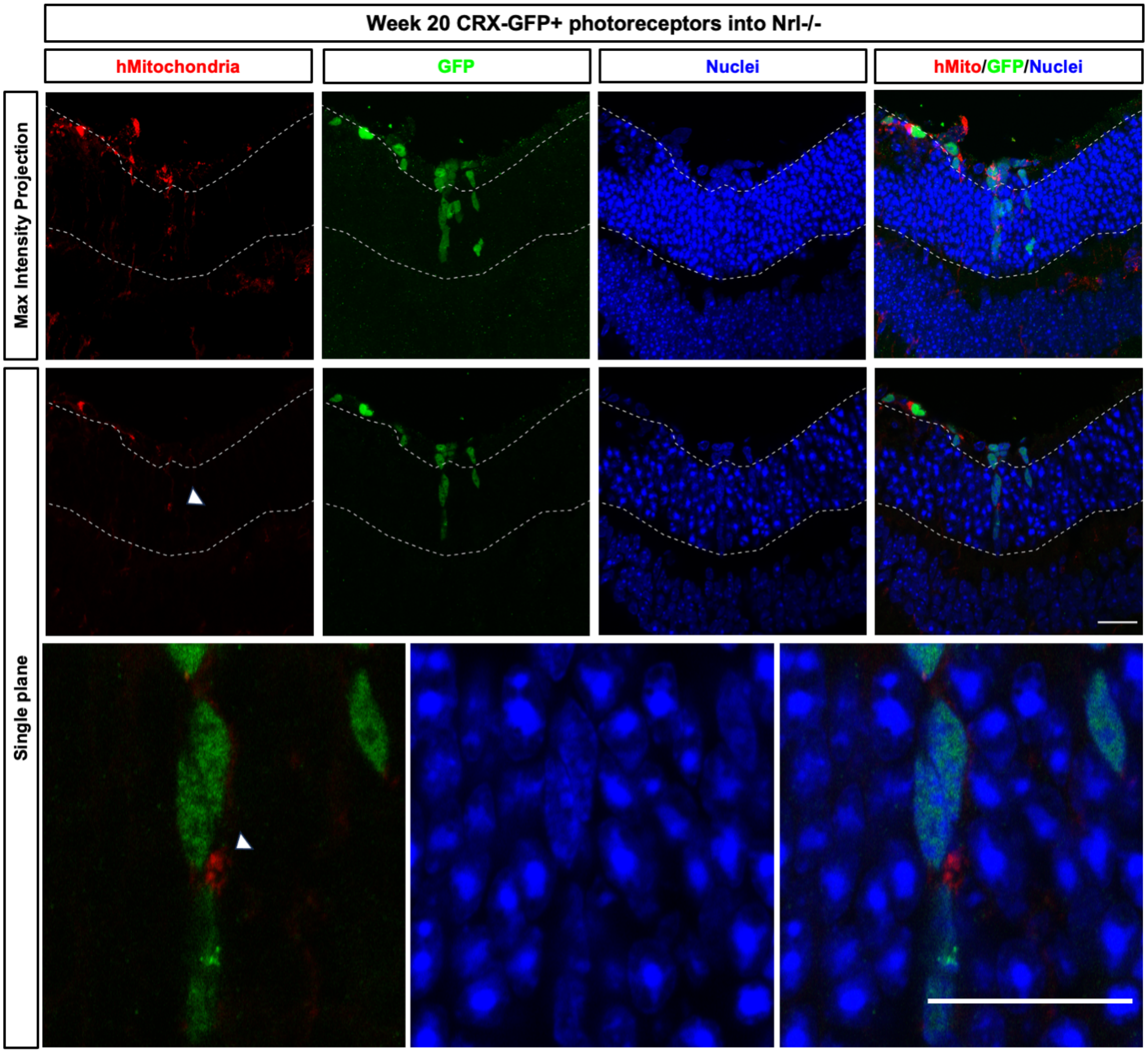

Supplemental Figure 4. **Week 20 CRX-GFP+ photoreceptors transplanted into *Nrl*$^{-/-}$ mouse retina integrate after 21 d.** Maximum intensity projection and single plane images with human mitochondria (red), CRX-GFP (green), and Hoechst (blue). Scale bar = 20 µm.

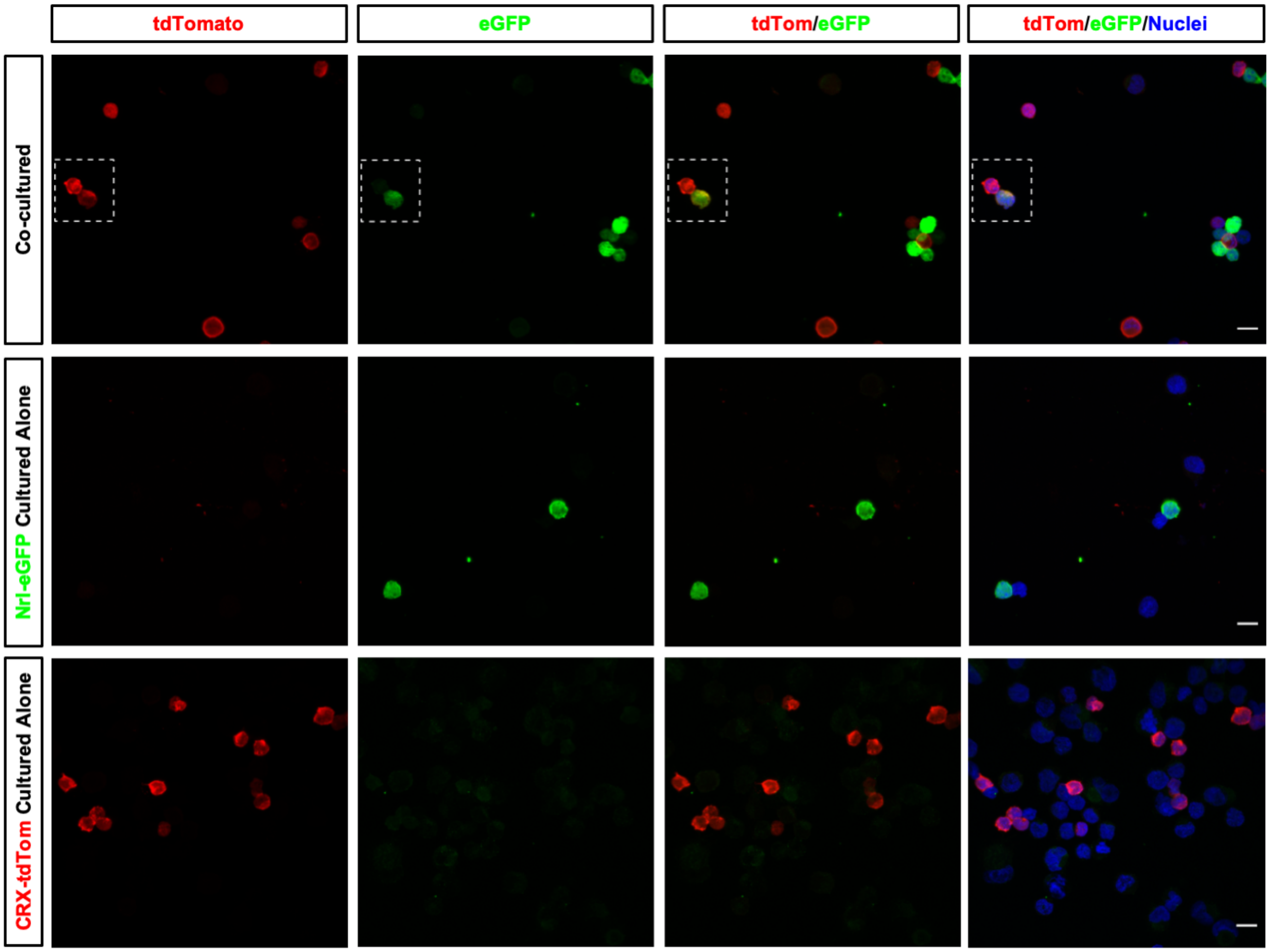

Supplemental Figure 5. Double positive cells were detected by immunofluorescence. After 7 days, the co-cultures were lifted with papain and stained with DAPI as a viability marker. The cells were enriched for photoreceptors using FACS to sort for either tdTomato OR eGFP. To prepare for IHC, the cells were plated after FACS on PDL/laminin coated dishes and fixed after 1 hour. Dissociated Nrl-eGFP organoids and dissociated CRX-tdTomato organoids were cultured alone and plated (unsorted) as imaging controls. Scale = 10 μm.

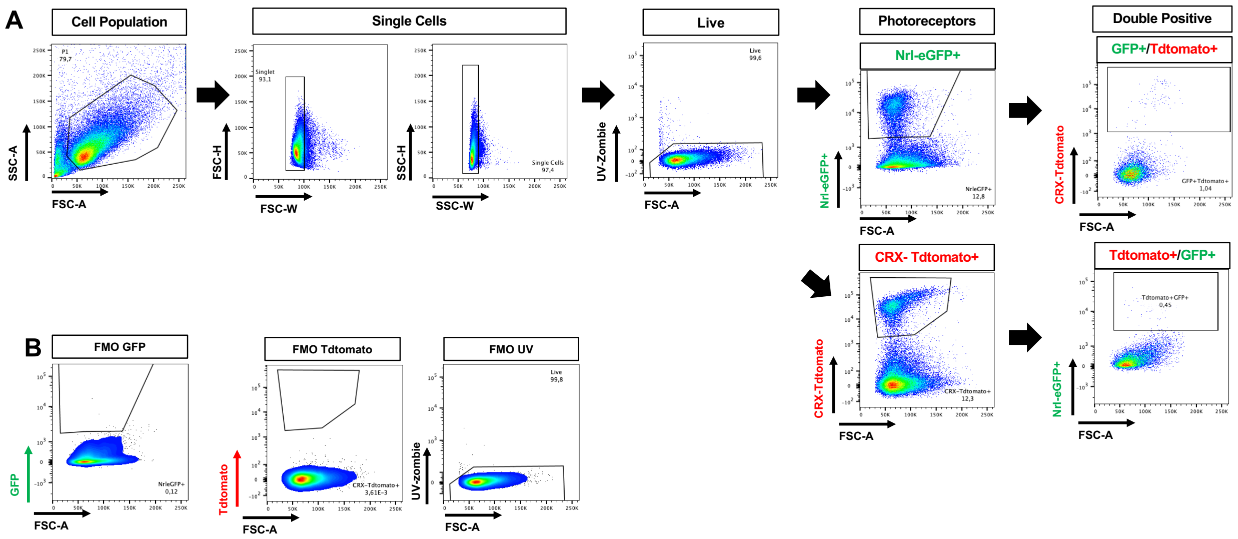

Supplemental Figure 6. **A**. Gating strategy for dissociated human retinal organoid and mouse retinal dissociates. P1 (gated FSC-A and SSC-A) is gated for single cells (FSC-H by FSC-W followed by SSC-H and SSC-W). Live/dead discrimination (UV-zombie negative) cells were gated. Photoreceptors were identified by either Nrl-eGFP or CRX-tdTomato gating and then the respective co-cultured population to obtain the proportion of double positive (Nrl-eGFP+/tdTomato+) population. **B**. Fluorescence minus one controls for the cytoplasmic protein co-cultures.

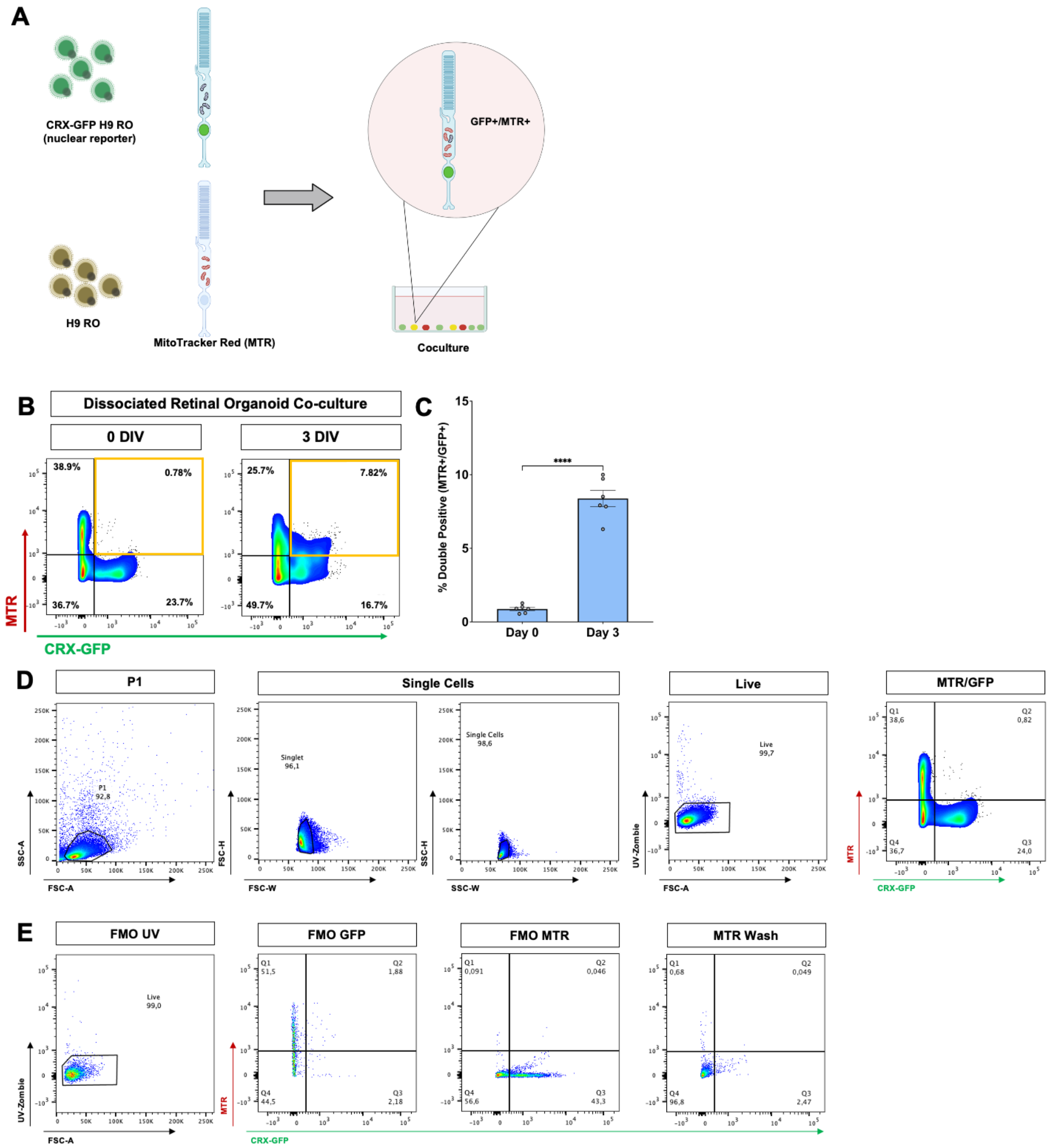

Supplemental Figure 7. Human photoreceptor cells from dissociated retinal organoids can transfer mitochondria. A. Schematic illustrating the *in vitro* co-culture setup. Week 20 retinal organoids (H9 derived) were dissociated, stained with Mitotracker Red (donor population), and co-cultured with dissociated week 20 CRX-GFP H9 retinal organoids (recipient population). Cells were harvested after 3 d of *in vitro* culture to quantify the proportion of double positive cells, which is indicative of CRX-GFP+ photoreceptor receiving mitochondria. **B.** Representative flow cytometry plot of live cells gated for MTR and the CRX-GFP+ reporter after days 0 and 3 of *in vitro* culture. **C**. Quantification of double positive (MTR+/GFP+) cells indicating transferred mitochondria from the MTR-stained retinal organoids dissociates into the CRX-GFP+ acceptor cells. Data presented as mean ± SEM (n = 6 biological replicates) analyzed by unpaired, 2-tailed t-test. **D.** Gating tree used for analysis. **E**. Fluorescence minus one and Mitotracker Red wash controls.

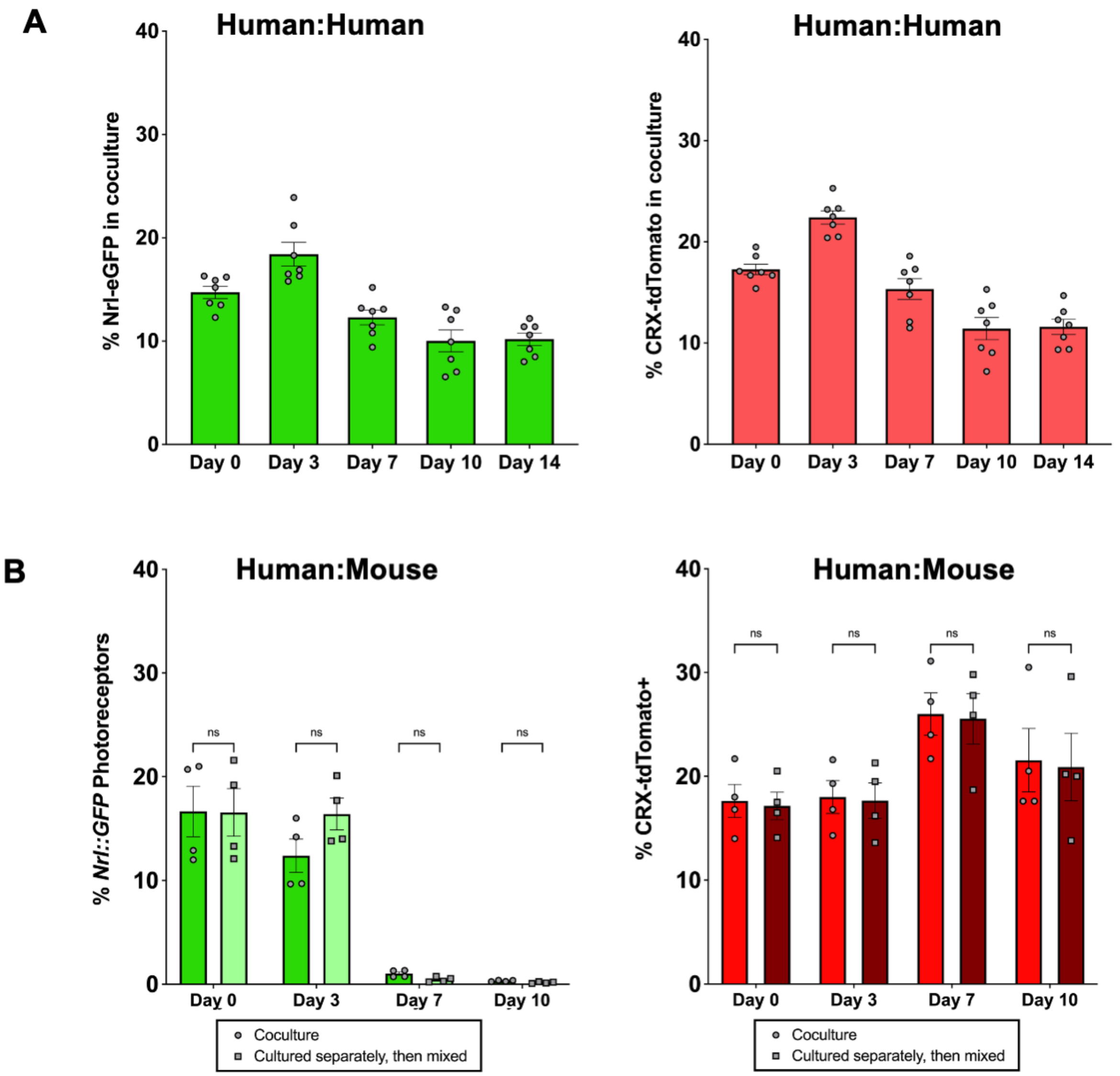

Supplemental Figure 8. Quantification of live photoreceptors in dissociated retinal organoid cocultures over time. A. Percentage of live human Nrl-eGFP+ and human CRX-tdTomato+ photoreceptors in human:human co-cultures. **B**. Mouse *Nrl::GFP+* photoreceptors survive poorly when cultured with human cells or in isolation. Percentage of mouse *Nrl::GFP+* photoreceptors and human CRX-Tdtomato+ photoreceptors in human:mouse co-cultures. Control samples cultured alone were mixed right before flow cytometry acquisition to achieve a baseline “no transfer” reading. mean ± SEM (n = 4-6 biological replicates).
